# Impact of a COVID-19 National Lockdown on Integrated Care for Hypertension and HIV

**DOI:** 10.5334/gh.928

**Published:** 2021-02-04

**Authors:** Jeremy I. Schwartz, Martin Muddu, Isaac Kimera, Mary Mbuliro, Rebecca Ssennyonjo, Isaac Ssinabulya, Fred C. Semitala

**Affiliations:** 1 Section of General Internal Medicine, Yale University School of Medicine, New Haven, CT, US; 2 Uganda Initiative for Integrated Management of Non-Communicable Diseases, Kampala, UG; 3 Makerere University Joint AIDS Program, Makerere University College of Health Sciences, Kampala, UG; 4 Uganda Heart Institute, Mulago National Referral Hospital, Kampala, UG; 5 Department of Medicine, Makerere University College of Health Sciences, Kampala, UG

**Keywords:** Hypertension, HIV, Access to care, Access to medicines, COVID-19

## Abstract

Research Letter Introduction: Measures to limit the spread of COVID-19, such as movement restrictions, are anticipated to worsen outcomes for chronic conditions such as hypertension (HTN), in part due to decreased access to medicines. However, the actual impact of lockdowns on access to medicines and HTN control has not been reported. Between March 25 and June 30, 2020, the Government of Uganda instituted a nationwide lockdown. Health facilities remained open, however motor vehicle transportation was largely banned. In Ugandan public health facilities, HTN services are offered widely, however the availability of HTN medicines is generally low and inconsistent. In contrast, antiretrovirals for people with HIV (PWH) are free and consistently available at HIV clinics. We sought to evaluate the impact of the lockdown on access to medicines and clinical outcomes among a cohort of Ugandan patients with HTN and HIV.

## Introduction

Measures to limit the spread of COVID-19, such as movement restrictions, are anticipated to worsen outcomes for chronic conditions such as hypertension (HTN) [1], in part due to decreased access to medicines [2]. However, the actual impact of lockdowns on access to medicines and HTN control has not been reported. Between March 25 and June 30, 2020, the Government of Uganda instituted a nationwide lockdown [3]. Health facilities remained open; however, motor vehicle transportation was largely banned. In Ugandan public health facilities, HTN services are offered widely; however, the availability of HTN medicines is generally low and inconsistent [4,6]. In contrast, antiretrovirals for people with HIV (PWH) are free and consistently available at HIV clinics. We sought to evaluate the impact of the lockdown on access to medicines and clinical outcomes among a cohort of Ugandan patients with HTN and HIV.

## Methods

Since August 2019, we have provided HTN/HIV services at the largest HIV clinic in Uganda, serving 16,500 PWH, through a prospective cohort study of integrated care delivery. All PWH who obtain care in this clinic are routinely screened for HTN. Those diagnosed with HTN are offered enrollment in the study and entered into a digital study registry. Contrary to the HIV-only care provided in most Ugandan HIV clinics, within this context, both conditions are managed by the same provider during the same appointment. We define HTN/HIV control as a last measured blood pressure (BP) less than 140/90mmHg and an undetectable HIV-1 viral load, respectively. HTN care is facilitated by a WHO HEARTS [5]-adapted treatment protocol that includes lifestyle advice and a stepped approach to prescribing of three anti-hypertensive medicines: amlodipine, valsartan, and hydrochlorothiazide. Medicines for both conditions are dispensed at no cost to participants. Most participants are seen monthly, but those who achieve dual control are seen every three months and prescribed medicines accordingly. During lockdown, the clinic remained open and medicine supply was consistent. However, access to these services was affected by travel restrictions and infection prevention measures that limited clinical care aside from medicine refills. Participants were encouraged to refill medicines in person and, if unable, were encouraged to obtain HTN/HIV care from health facilities closer to home. Appointments were considered missed if participants did not present for a medicine refill within seven days of a scheduled appointment. Participants who miss appointments are routinely contacted by phone and asked whether they had obtained care or medicines at another health facility. The study was approved by The AIDS Support Organization (TASO) Institutional Review Board, Kampala, Uganda.

## Results

By end of March 2020, we had enrolled 1,133 participants, of whom 72% had achieved HTN control. HIV control was consistently above 97% among the cohort. In the three months preceding lockdown (pre-lockdown), the percentage of missed appointments ranged from 0.4–5.2%, while in the three months of lockdown, this ranged from 16.2%–21.5%. Pre-lockdown, no participants who missed appointments sought HIV and/or HTN services elsewhere. During lockdown, 49–66% of those who missed appointments sought care at other health facilities, according to self-report by phone or at their subsequent visit. Of those who sought care elsewhere, 92–100% reported having been dispensed only HIV medicines, not HTN medicines (Table). Pre-lockdown, there was one participant death, which was not HTN related. During lockdown, there were three HTN-related deaths and one stroke. In July, the month immediately post-lockdown, 319 participants had BP measured, and 87 had viral load measured. Of this group, 56% had controlled BP compared to 68% pre-lockdown, while 99% had HIV control, compared to 98% pre-lockdown.

**Table:** Changes in missed appointments and access to hypertension and HIV medicines for study participants before and during the national lockdown (January–June 2020).

	January	February	March	April	May	June

Scheduled appointments	736	475	286	494	385	293
Missed appointments: n (%)	25 (3.4)	2 (0.4)	15 (5.2)	80 (16.2)	83 (21.5)	51 (17.4)
Obtained HIV and/or HTN services from other health facilities	0 (0)	0 (0)	0 (0)	53 (66)	49 (59)	25 (49)
Obtained only HIV medicines from other health facilities: n (%)	n/a	n/a	n/a	49 (92)	47 (96)	25 (100)
Obtained HIV *and* HTN medicines from other health facilities: n (%)	n/a	n/a	n/a	4 (8)	2 (4)	0 (0)

Abbreviation: HTN = hypertension.

## Discussion

Lockdowns to limit the spread of COVID-19 present a novel barrier to obtaining medicines for chronic conditions. Though HIV medicines were nearly universally obtained during this period, HTN medicines were not due to widespread underdevelopment of integrated service delivery for chronic conditions. This resulted in worsened HTN control and an increase in HTN-related adverse events, while HIV control was unaffected. Persons living with multiple chronic conditions require consistent access to all their medicines, even during times of widespread health crises. The implications of lockdowns on medicine access for persons living with chronic conditions must be considered as efforts to contain COVID-19 continue globally.

## References

[1] Lim MA, Huang I, Yonas E, Vania R, Pranata R. A wave of non-communicable diseases following the COVID-19 pandemic. Diabetes Metab Syndr. 2020; 14(5): 979–980. DOI: 10.1016/j.dsx.2020.06.050 32610263 PMC7318943

[2] Ghosal S, Sinha B, Majumder M, Misra A. Estimation of effects of nationwide lockdown for containing coronavirus infection on worsening of glycosylated haemoglobin and increase in diabetes-related complications: A simulation model using multivariate regression analysis. Diabetes Metab Syndr. 2020; 14(4): 319–323. DOI: 10.1016/j.dsx.2020.03.014 32298984 PMC7146694

[3] Uganda orders two-week lockdown to contain Coronavirus. Agence France-Presse. https://www.courthousenews.com/uganda-orders-two-week-lockdown-to-contain-coronavirus/. Accessed August 20, 2020.

[4] Armstrong-Hough M, Kishore SP, Byakika S, Mutungi G, Nunez-Smith M, Schwartz JI. Disparities in availability of essential medicines to treat non-communicable diseases in Uganda: A Poisson analysis using the Service Availability and Readiness Assessment. PLoS One. 2018; 13(2): e0192332 DOI: 10.1371/journal.pone.0192332 29420640 PMC5805288

[5] HEARTS. Technical package for cardiovascular disease management in primary health care Geneva: World Health Organization; 2016.

[6] Armstrong-Hough M, Sharma S, Kishore SK, Schwartz JI. Variation in the availability and cost of essential medicines for non-communicable diseases in Uganda: a descriptive time-series analysis. PLoS ONE. 2020; 15(12): e0241555 DOI: 10.1371/journal.pone.0241555 33362249 PMC7757794

